# RETRACTION: CXCL13 Promotes Osteogenic Differentiation of Mesenchymal Stem Cells by Inhibiting miR‐23a Expression

**DOI:** 10.1155/sci/9875419

**Published:** 2026-01-25

**Authors:** Stem Cells International

RETRACTION: F. Tian, X.‐L. Ji, W.‐A. Xiao, B. Wang, and F. Wang, “CXCL13 Promotes Osteogenic Differentiation of Mesenchymal Stem Cells by Inhibiting miR‐23a Expression,” *Stem Cells International*, 2015, 632305, https://doi.org/10.1155/2015/632305.

The above article, published online on 16 February 2015 in Wiley Online Library (wileyonlinelibrary.com), has been retracted by agreement between the journal Associate Editor, James Adjaye, and John Wiley & Sons Ltd. The retraction has been agreed due to inconsistencies identified in Figures 1 and 3 [1]. Specifically:

• There are unexpectedly repeated regions between the control and CXCL13 panels in Figure 1a
• There are unexpectedly repeated regions between the CXCL13 panel in Figure 1a and the NC + CXCL13 panel in Figure 3a
• There are unexpectedly repeated regions between the CXCL13 panel in Figure 1a and the miR‐23a mimic + CXCL13 panel in Figure 3a
• There are unexpectedly repeated regions within the NC + CXCL13 panel in Figure 3a

The authors did not respond when asked for clarification. After assessment of the concerns and confirmation from the editorial board, the results are deemed unreliable and the article is retracted.
